# Formation pathways of mesoporous silica nanoparticles with dodecagonal tiling

**DOI:** 10.1038/s41467-017-00351-8

**Published:** 2017-08-15

**Authors:** Yao Sun, Kai Ma, Teresa Kao, Katherine A. Spoth, Hiroaki Sai, Duhan Zhang, Lena F. Kourkoutis, Veit Elser, Ulrich Wiesner

**Affiliations:** 1 Department of Materials Science and Engineering, Cornell University, Ithaca, NY 14853 USA; 2 School of Applied and Engineering Physics, Cornell University, Ithaca, NY 14853 USA; 3 Kavli Institute at Cornell for Nanoscale Science, Ithaca, NY 14853 USA; 4 Department of Physics, Cornell University, Ithaca, NY 14853 USA; 5 Simpson Querrey Institute for Bionanotechnology, Northwestern University, Evanston, IL 60201 USA

## Abstract

Considerable progress in the fabrication of quasicrystals demonstrates that they can be realized in a broad range of materials. However, the development of chemistries enabling direct experimental observation of early quasicrystal growth pathways remains challenging. Here, we report the synthesis of four surfactant-directed mesoporous silica nanoparticle structures, including dodecagonal quasicrystalline nanoparticles, as a function of micelle pore expander concentration or stirring rate. We demonstrate that the early formation stages of dodecagonal quasicrystalline mesoporous silica nanoparticles can be preserved, where precise control of mesoporous silica nanoparticle size down to <30 nm facilitates comparison between mesoporous silica nanoparticles and simulated single-particle growth trajectories beginning with a single tiling unit. Our results reveal details of the building block size distributions during early growth and how they promote quasicrystal formation. This work identifies simple synthetic parameters, such as stirring rate, that may be exploited to design other quasicrystal-forming self-assembly chemistries and processes.

## Abstract (web summary)

Probing the growth pathways of quasicrystalline materials, where tiling units arrange with local but no long-range order, remains challenging. Here, the authors demonstrate that dodecagonal tiling of mesoporous silica nanoparticles occurs via irreversible packing of micelles with non-uniform size distribution.

## Introduction

Quasicrystals exhibit highly ordered local structure but lack long-range translational periodicity, and permit symmetry operations that are forbidden in classical crystallography. Since the discovery of quasicrystalline order in metal alloys^1^, quasicrystals have been observed in a wide variety of materials, including thin films^2^, liquid crystals^3^, polymers^4^, colloids^5^, and mesoporous networks^6^, demonstrating that quasicrystallinity can be considered a universal form of ordering.

Advances in the identification of quasicrystalline materials have attracted widespread attention to the underlying mechanisms that govern quasicrystal formation. In self-assembled micellar systems, access to quasicrystal and quasicrystal approximant phases depends on building block geometry, controlled in recent experimental studies through molecular design of giant surfactant molecules^7, 8^ and asymmetric diblock copolymers^9, 10^. These experimental findings are consistent with simulations of self-assembled systems, where building blocks designed with substantial shape polydispersity or patchy surfaces promote quasicrystalline order^11, 12^. In metal alloys, studies of quasicrystal formation have verified theoretical predictions that quasicrystalline order is maintained during grain growth by a local error-and-repair type mechanism^13, 14^. Despite these successes, experimental investigations of early quasicrystal formation mechanisms remain challenging, largely due to a lack of chemistries that facilitate direct observation.

Mesoporous silica materials with pore sizes in the range between 2 and 50 nm have attracted widespread attention due to their precisely tunable macroscopic form, chemical functionality, and mesopore structure^15, 16^. There have been reports of mesoporous silica bulk materials^15–18^ and nanoparticles^19–21^ with a variety of pore structures, including hexagonal^16^, cubic^20^, and quasicrystalline structures^6, 20, 22^. The formation of mesoporous silica is a result of silica condensation directed by molecular templates, such as surfactants or polymers^16, 23^. This cooperative assembly process is driven by non-directional interactions between micelles and silica precursors, making mesoporous silica similar to systems of metal alloys, where non-directional bonding plays a key role in achieving the non-periodic bonding geometries required for quasicrystal formation.

Inspired by this analogy, we have synthesized surfactant-directed mesoporous silica nanoparticles (MSNs) with dodecagonal tiling and used them as a test bed for studying early formation mechanisms. While previous reports of quasicrystalline mesoporous silicas have used anionic surfactants^6, 22^, we demonstrate that such materials can also be synthesized by self-assembly of positively charged surfactant micelles. The dodecagonal quasicrystal structure is the last in a sequence of four different structures discovered in this study as a function of either micelle pore expander concentration or reaction stirring rate. Via direct visualization employing cryo-transmission electron microscopy (cryo-TEM) and quantitative image analysis, we find that quasicrystallinity is predominantly governed by asymmetries in the micelle size distribution and identify micelle pore expander concentration and reaction stirring rate as equivalent experimental control parameters. Furthermore, precise control of the silica chemistry enables MSN sizes to be tuned from above 100 nm, where dodecagonal tiling patterns are well developed, to below 30 nm, where particles consisting of only a single triangle or square tiling unit represent the earliest formation stages of the dodecagonal tiling. Experimental results are then compared to growth simulations. Our results suggest that the incorporation of building blocks with asymmetric size distributions at early times in the growth process promotes quasicrystal formation and that simple synthesis parameters like stirring rate can be used for their control.

## Results

### [TMB]-induced transitions between four MSN structures

**Fig. 1:**
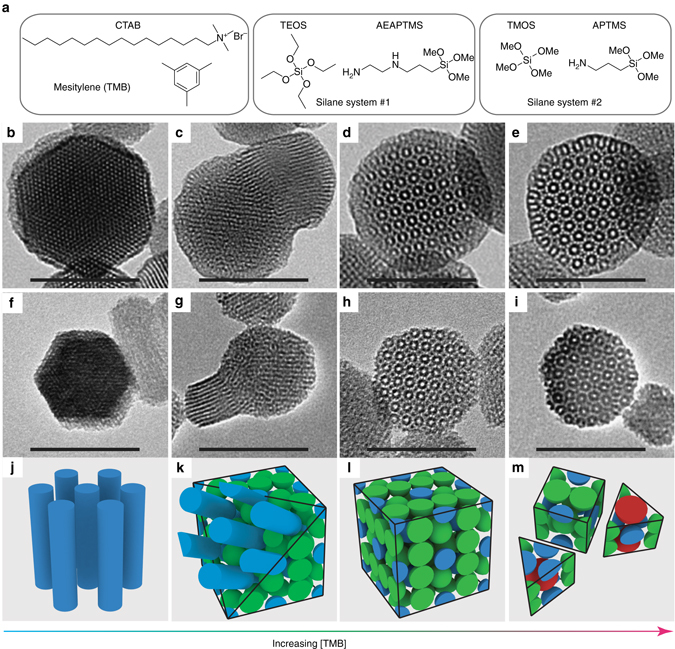
Four MSN structures observed in two silane systems as a function of mesitylene concentration. **a** Chemical structures of key reagents used in TEOS/AEAPTMS and TMOS/APTMS synthesis systems. **b**–**e** TEM images of MSNs prepared from TEOS/AEAPTMS with 12 mM (**b**), 29 mM (**c**), 47 mM (**d**), and 116 mM (**e**) mesitylene (TMB), using constant ammonium hydroxide concentration (13.8 mM) and stirring rate (650 rpm). **f**–**i** TEM images of MSNs prepared from TMOS/APTMS with 4 mM (**f**), 10 mM (**g**), 14 mM (**h**), and 72 mM (**i**) TMB, using constant ammonium hydroxide concentration (150 mM) and stirring rate (600 rpm). **j**–**m** Micelle packing models corresponding to MSNs in **b**–**i**. All *scale bars* are 100 nm

MSNs were synthesized by co-condensing a mixture of silane precursors, tetraethyl orthosilicate (TEOS) and N-(2-aminoethyl)-3-aminopropyltrimethoxysilane (AEAPTMS), in the presence of hexadecyltrimethylammonium bromide (CTAB) surfactant micelles (see molecular structures in Fig. 1a). The aminosilane was added to tailor the packing behavior of the CTAB surfactant molecules^24, 25^, enabling access to more complex silica nanostructures^19, 20, 26^. The CTAB micelle size and size distribution was systematically varied by adjusting the concentration of a micelle pore expander, mesitylene (TMB)^27–29^. TEM images show that as the concentration of TMB, [TMB], was increased, the MSN structure changed from hexagonal (Fig. 1b) to multicompartment (Fig. 1c and Supplementary Fig. 1) to cubic (Fig. 1d) and finally to a structure with dodecagonal symmetry (Fig. 1e).

Small-angle X-ray scattering (SAXS) measurements, which average over macroscopic volumes, were performed on the TEOS/AEAPTMS particles (Supplementary Fig. 2). The results corroborate the structural transitions observed by TEM (Fig. 1b–e). At 12 mM [TMB], the SAXS pattern shows reflections consistent with hexagonal symmetry. When [TMB] is increased to 29 mM, a well-resolved set of cubic *Pm* $\bar 3$ *n* reflections from the particle cores is superposed with a prominent set of hexagonal *p*6*mm* reflections from the particle arms. This pattern is consistent with previously reported SAXS measurements from multicompartment MSNs^19^. At 47 mM [TMB], the SAXS pattern shows reflections consistent with a cubic particle with *Pm* $\bar 3$ *n* lattice^24^. Expected peak positions for cubic *Pm* $\bar 3$ *n* and hexagonal *p*6*mm* are indicated in the SAXS patterns using *solid* and *dashed lines*, respectively (Supplementary Fig. 2). As [TMB] is further increased to 116 mM and beyond, the SAXS patterns lose more and more features and lattices cannot unambiguously be assigned. TEM tilt series (Supplementary Fig. 3) as well as TEM tomography failed to establish the three-dimensional (3D) particle structure, the latter as a result of severe beam damage of the organic-silica hybrids during data acquisition.

To deconvolute the effects of [TMB] from the specifics of the silica chemistry, we examined a second, independent organosilane system, composed of tetramethyl orthosilicate (TMOS) and (3-aminopropyl)trimethoxysilane (APTMS) (Fig. 1a). MSNs synthesized using TMOS and APTMS showed similar transitions as a function of increasing [TMB] (Fig. 1f–i). Comparison between the TEOS/AEAPTMS and TMOS/APTMS systems suggests that the observed structural transitions do not depend on the specifics of the silane and aminosilane molecules used here. However, it is important to note that the presence of an aminosilane is critical for the formation of quasicrystalline MSNs (Supplementary Fig. 4a, b). This observation is consistent with our expectation that the aminosilane mediates changes in the packing behavior of the CTAB surfactant^24, 25^, facilitating the formation of more complex nanostructures^19, 20, 26^.

**Fig. 2:**
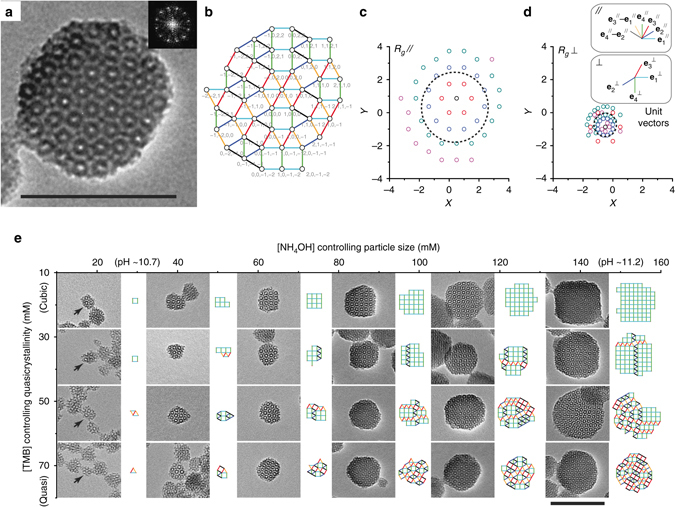
Quasicrystallinity of TMOS/APTMS-derived MSNs. **a** TEM image of a MSN with dodecagonal symmetry, synthesized using TMOS and APTMS. The *scale bar* is 100 nm. The insert in **a** shows a diffractogram of the TEM image exhibiting 12-fold symmetry. **b** Square-triangle tiling and parallel space coordinates of the nanoparticle shown in **a**. Edges with different colors correspond to the six dodecagonal directions in parallel space. The corresponding dodecagonal directions in parallel and perpendicular space are shown in **d**. **c**, **d** Parallel and perpendicular space coordinates for the *triangle-square* tiling shown in **b**. *Dotted circles* with radius equal to *R*_*g*_// and *R*_*g*_⊥ are shown on the parallel and perpendicular space plots, respectively. The colors in **c** and **d** show how individual points move during the transformation. **e** TEM images and corresponding square-triangle tilings of representative MSNs (*arrows*) synthesized from different concentrations of mesitylene (TMB) and ammonium hydroxide (NH_4_OH). The stirring rate was 600 rpm for all syntheses. All images have the same magnification; *scale bar* is 100 nm

Pore patterns of MSNs like the ones in Fig. 1e were tiled using squares and equilateral triangles (Fig. 2a, b). These MSNs exhibit two distinctive features of dodecagonal quasicrystals. Fast Fourier transform (FFT) analysis of TEM images reveals 12-fold symmetry and no translational periodicity is observed in the two-dimensional (2D) tiling patterns extracted from the TEM images (Fig. 2a, b)^11^. The tile edges correspond to dodecagonal directions and each vertex can be described by ${{\bf r}^{{\rm{/}}\!{\rm{/}}}} = {n_1}{\bf e}_1^{{\rm{/}}\!{\rm{/}}} + {n_2}{\bf e}_2^{{\rm{/}}\!{\rm{/}}} + {n_3}{\bf e}_3^{{\rm{/}}\!{\rm{/}}} + {n_4}{\bf e}_4^{{\rm{/}}\!{\rm{/}}}$ (Fig. 2b). Each vertex in parallel (Fig. 2c) space corresponds to a unique vertex in perpendicular (Fig. 2d) space^30^. Figure 2c and d show by color how individual points move during this transformation. A comparison of the radii of gyration in perpendicular and parallel space, *R*_*g*_⊥ vs. *R*_*g*_//, is a measure of the quasicrystallinity of a pattern: the more a pattern shrinks, the more quasicrystalline it is (compare patterns in Fig. 2c–d).

### Synthesis of MSNs with varying size and quasicrystallinity

We systematically investigated the growth of dodecagonal tilings in mesoporous silica all the way down to a single tiling unit by capitalizing on well-established silica chemistry to control the particle size. The comparatively fast hydrolysis of TMOS relative to TEOS facilitated access to smaller MSNs^21^. Precise particle size control in this system was further achieved using variations in reaction pH, where the increasing condensation rate associated with lowered pH resulted in smaller particle sizes^31^. In this way, MSNs could be synthesized with sizes <30 nm such that associated tilings were composed of a single triangle or square tiling unit. TEM images of quasicrystalline MSNs, synthesized using constant [TMB] and varying ammonium hydroxide concentration, [NH_4_OH], are shown in the *bottom row* of Fig. 2e. Interestingly, qualitative assessment of the triangle-square tilings extracted from these images suggests that quasicrystallinity does not vary with changes in reaction pH.

Increasing [TMB] from 14 to 72 mM introduces a gradual transition from cubic to quasicrystalline pore structure, where triangle-square tilings extracted from MSNs synthesized using intermediate [TMB] are mixed phase rather than purely cubic or quasicrystalline. Tuning reaction pH at low and intermediate [TMB] again resulted in particle sizes ranging from >100 nm down to <30 nm with no observable pH-dependent variations in pore structure. Figure 2e illustrates the range of particle structures obtained from over ~50 synthesis batches of particles (see also Supplementary Fig. 4).

### Quantitative analysis of micelle size distributions

**Fig. 3:**
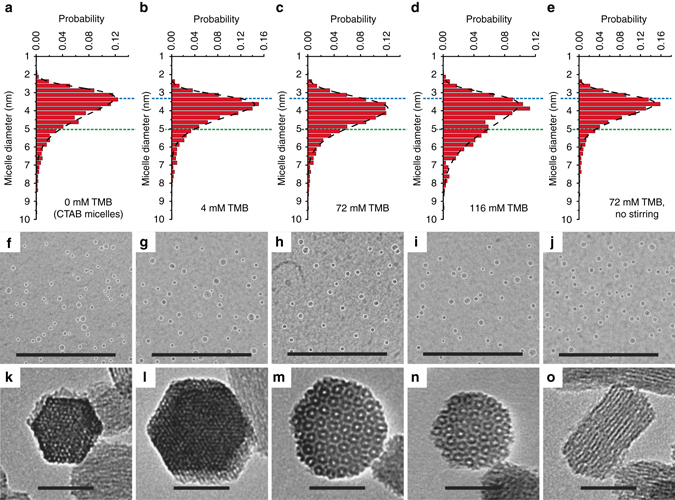
Cryo-TEM of native micelle solutions prepared using varying mesitylene concentrations and stirring rates. **a**–**e** Micelle size distributions before silane addition, prepared using 0 mM (**a**), 4 mM (**b**), 72 mM (**c**), and 116 mM (**d**) mesitylene (TMB) under constant stirring (600 rpm) and 72 mM (**e**) TMB with no stirring. The ammonium hydroxide concentration was kept constant (150 mM) across all solutions. Distribution fits are shown using *black dashed lines*. *Blue dashed lines* indicate the peak position of the micelle size distribution of CTAB micelles without TMB and *green dashed lines* are shown as an additional reference for comparison across different plots. Comparison between the micelle size distributions demonstrates that although the peak position does not vary substantially, the combination of increased TMB concentration and mechanical agitation causes a shift to larger average micelle diameters and an increase in the frequency of large micelles with sizes above 5 nm. **f**–**j** Cryo-TEM images of the micelle solutions before silane addition corresponding to conditions used in **a**–**e**, respectively. Individual micelles are identified using a self-written python code and are circled in *white*. **k**–**o** TEM images of MSNs synthesized via adding TMOS and APTMS into micelle solutions corresponding to conditions used in **a**–**e**, respectively. *Scale bars* in both sets of images are 50 nm

Although the observed MSN structures result from cooperative interactions between the CTAB micelles and the silica species^32^, the above observations point to the starting micelle size distribution as a key factor in the formation of quasicrystalline MSNs. Recent reports provide supporting evidence for the role of building block polydispersity in self-assembly, demonstrating that particles with broad monomodal or bimodal size distributions form more complex structures^7, 8, 33^. We have characterized the starting micelle sizes using cryo-TEM (Fig. 3). Representative micelle size distributions from native CTAB/TMB solutions prepared using increasing [TMB] and constant stirring rate are shown in Fig. 3a–d. Micelle solutions prepared without TMB or using low [TMB] (4 mM) led to formation of hexagonal MSNs (Fig. 3k, l), whereas use of high [TMB] (72 and 116 mM) resulted in quasicrystalline MSNs (Fig. 3m, n). Comparison of the entire series reveals that increasing [TMB] causes a shift to larger average micelle diameter as well as an increase in the positive skewness of the micelle size distribution, e.g., compare numbers of micelles with sizes above 5 nm as [TMB] increases (Fig. 3a–d).

Cryo-TEM experiments further demonstrated that mechanical agitation (stirring), in addition to TMB, is a requirement for producing these broadened micelle size distributions (Fig. 3e, j, o), consistent with recent observations of complex polymer micelle size distributions in the presence of a co-solvent and solution agitation^34^. Associated structural transitions are illustrated schematically by the micelle packings in Fig. 1j–m ^6, 35, 36^, using a model with three discrete micelle sizes for simplicity. Hexagonal MSNs have channel-like pores templated by elongated CTAB micelles (*blue*) (Fig. 1j). Multicompartment MSNs are composed of a *Pm* $\bar 3$ *n* cubic core connected epitaxially at the (111) face to a hexagonal structure^19^. The *Pm* $\bar 3$ *n* cubic core is composed of two types of spherical micelles, one of which is slightly larger (*green*) than the other (*blue*) due to increased TMB loading (Fig. 1k). As [TMB] is further increased, subsequently single phase *Pm* $\bar 3$ *n* cubic MSNs (Fig. 1l) and MSNs with dodecagonal symmetry are formed, the latter incorporating even larger micelles (*red*) (Fig. 1m).

These observations are supported by pore size analysis of nitrogen sorption measurements on TEOS/AEAPTMS particles after CTAB removal that show marked pore size increase and broadening of the pore size distribution with increasing [TMB] (Supplementary Fig. 2). At 12 mM (hexagonal MSNs), the pore size distribution is well fit by a single log-normal distribution (Supplementary Fig. 5). However, as [TMB] is increased to 47 and 116 mM (cubic and quasicrystalline MSNs), the increasingly broadened pore size distributions can no longer be described by unimodal log-normal fits, suggesting that additional micelle populations may be involved in the formation processes of these more complex structures. As a first approximation, these data are therefore fit using a superposition of two and three log-normal distributions, respectively (Supplementary Fig. 5). Average pore sizes extracted from these fits (Supplementary Table 1) support the hypothesis that the structural changes observed with increasing [TMB] are due to the presence of additional, larger micelle populations. Additional experiments demonstrated that the same structural transitions and pore size increases were achieved using constant [TMB] (29 mM) but increased stirring rates that also affects micelle size (Supplementary Figs. 5, 6).

### Tiling analysis

**Fig. 4:**
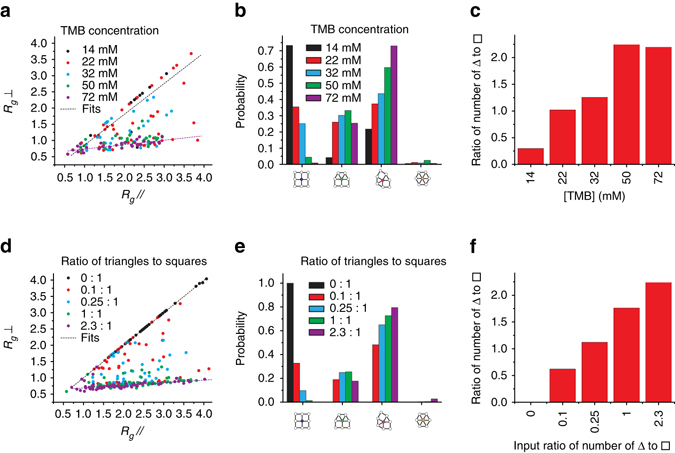
Analysis of TMOS/APTMS-derived MSNs and simulated structures. **a** Plot of *R*_*g*_ in perpendicular vs. parallel space, calculated for MSNs synthesized from five different mesitylene (TMB) concentrations. **b** Distribution of pore conformers (4^4^, 3^3^4^2^, 3^2^4^1^3^1^4^1^, and 3^6^) calculated for particles synthesized from five different TMB concentrations. **c** Ratio of triangle to square tiles calculated for particles in **a**. **d**–**f** Plots corresponding to **a**–**c** for 500 particles derived from simulations with five different triangle to square ratios

The MSNs shown in Fig. 1e, i exhibit indicators of quasicrystallinity (i.e., 12-fold symmetric diffractogram and no translational periodicity). Since there are no local constraint rules that enforce quasicrystallinity in systems of triangles and squares, quasicrystallinity in these finite structures is instead characterized by statistical analysis of the phason coordinates. We analyzed the tilings extracted from the TMOS/APTMS-derived MSNs synthesized as a function of increasing [TMB] (Fig. 2e and Supplementary Fig. 5). Figure 4a shows a plot of *R*_*g*_⊥ vs. *R*_*g*_// calculated from the tilings of individual MSNs synthesized with systematic variations in [TMB] and [NH_4_OH]. The slope of the linear fit *R*_*g*_⊥ = *AR*_*g*_//* + B* provides the magnitude of the phason strain, a quantitative measure of quasicrystallinity^6, 30^. MSNs synthesized at the highest [TMB] (72 mM) and varying pH lie on the same line, which is well described by the fit with phason strain equal to 0.14 (see TEOS/AEAPTMS data in Supplementary Fig. 7) approaching the zero-phason strain of a dodecagonal quasicrystal^6^ and consistent with the phason behavior of a random tiling quasicrystal^30^. Additionally, the fit demonstrates that these MSNs have similar quasicrystallinity, which contrary to previous studies^6^, is independent of the reaction pH and pH-induced changes in the interaction potentials between micelles.

TEM images of MSNs synthesized using low [TMB] and varying pH (Fig. 2e and Supplementary Fig. 4) exhibit predominantly cubic structure. Interestingly, a few quasicrystalline MSNs are always found in a batch of cubic MSNs, resulting in a slope that is slightly smaller than 1, i.e., the phason strain of a perfectly cubic crystal. Comparison between MSNs synthesized using low and high [TMB] demonstrates that changes in quasicrystallinity are due to differences in [TMB] rather than pH. Particles synthesized at intermediate [TMB] between 22 and 50 mM exhibited substantial fluctuations in the plot of *R*_*g*_⊥ vs. *R*_*g*_// (Fig. 4a). More specifically, individual particles from a single batch of MSNs synthesized in this regime may be cubic, quasicrystalline, or mixed phase, suggesting that not only [TMB] and stirring rate but also other factors are at play.

From the tiling analysis the distribution of four fundamental geometrical conformations was derived for all batches/particles, i.e., 4^4^, 3^3^4^2^, 3^2^4^1^3^1^4^1^, and 3^6^, where 4 and 3 represent square and triangle base units and the exponent indicates the number of adjacent base units incident on a single vertex (Fig. 4b). This analysis shows that with increasing [TMB], cubic 4^4^ conformers decrease, 3^3^4^2^ conformers first increase but then roughly stay constant, and 3^2^4^1^3^1^4^1^ conformers increase. All MSN samples analyzed have low abundance of 3^6^ conformers. At the same time, the ratio of the overall numbers of triangles to squares increases with [TMB] (Fig. 4c). Considering that the triangle tile contains the largest micelles (Fig. 1m), these data imply that the presence of large micelles plays a key role in quasicrystal formation. The growth of quasicrystals may also be highly dependent on the dynamic packing process of differently sized micelles forming triangle and cubic tiles, as indicated by the substantial phason strain fluctuations of the MSNs synthesized at intermediate [TMB]. Simulations were carried out in order to elucidate the origin of these fluctuations and reveal dominant factors in this [TMB] regime.

### Simulated single-particle growth trajectories

Quasicrystal growth has previously been modeled in two dimensions using triangle-square tilings^6, 13, 37^. We have developed an irreversible MSN growth model that produces 2D tilings that mimic the observed features of our experimental system (see Supplementary Methods for details). Particle growth in these simulations proceeds via irreversible aggregation of square and equilateral triangle tiles. This accounts for experimental observations wherein packings of small and medium-sized micelles result in square tiles (*green* and *blue* in Fig. 1l) and the involvement of a third, larger micelle gives rise to triangle tiles (*red* in Fig. 1m). Each tile is randomly chosen from a weighted shape distribution given by an input triangle/square number ratio that reflects the increasing number of triangles observed in MSNs synthesized at increasing [TMB] (Fig. 4b, c). The probability of tile attachment on the growing cluster is governed by both shape and site-specific interaction parameters. We also include an additional threshold parameter that allows the system to choose replacement tiles if attachment probabilities above a minimum value do not exist. For each triangle/square ratio, several batches of particles with size distributions that approximately match those of experimentally observed particles were generated. Simulated particles were analyzed according to the methods outlined for the experimental particles. Although this is a simplified model, comparison of Fig. 4d through f with Fig. 4a through c shows that it was sufficient to reproduce the experimentally observed features. These results suggest that the structural transition observed is primarily a function of the triangle/square ratio or micelle size distribution in an irreversible, random growth process.

**Fig. 5:**
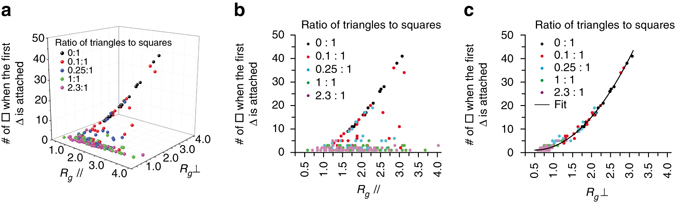
Analysis of simulated single-particle growth trajectories. **a** 3D plot showing that phason strain is highly dependent on the time at which the first triangle is added to the growing particle. **b**, **c** 2D projections of the 3D plot in **a**. The data in **c** is fitted using equation *Na* ^2^ = *AR*_*g*_⊥^2^ + *aBR*_*g*_⊥ + *a* ^2^ *C*, where *a* = 1 is the edge length of a square tile, *N* is the number of tiles, and *A*, *B*, and *C* are fitting parameters. This is consistent with the theoretical relationship between the area and the radius of gyration, *R*_*g*_, of a 2D square packing pattern (Supplementary Methods)

Analysis of simulated single-particle growth trajectories (Fig. 5a) shows the relationship between quasicrystallinity (*R*_*g*_⊥ vs. *R*_*g*_//) and the size of the particle or the number of square tiles present (plotted along the *z*-axis) when the first triangle is added. For perfect cubic particles, the total number of squares at the end of the simulation is counted instead. At the highest triangle/square input ratio of 2.3^38^, all data points (*purple*) are highly concentrated in a low *z*-range in Fig. 5a, suggesting that in this irreversible tiling process the early appearance of a first triangle contributes to the growth of highly quasicrystalline particles. By comparison, the appearance of the first triangle in particles with a triangle/square input ratio of 0.1 (*red*) is widely distributed in the growth process causing the spread in quasicrystallinity observed in Fig. 4d. 2D projections of Fig. 5a, which represent the dependence of *R*_*g*_// and *R*_*g*_⊥ on the timing (i.e., particle size) of the first triangle addition, are shown in Fig. 5b and c, respectively. In Fig. 5c, all data points fall onto a master curve, *N* = *N* (*R*_*g*_⊥), that relates the area of a 2D square tiling pattern (∝*N*) to its radius of gyration (see Supplementary Methods).

**Fig. 6:**
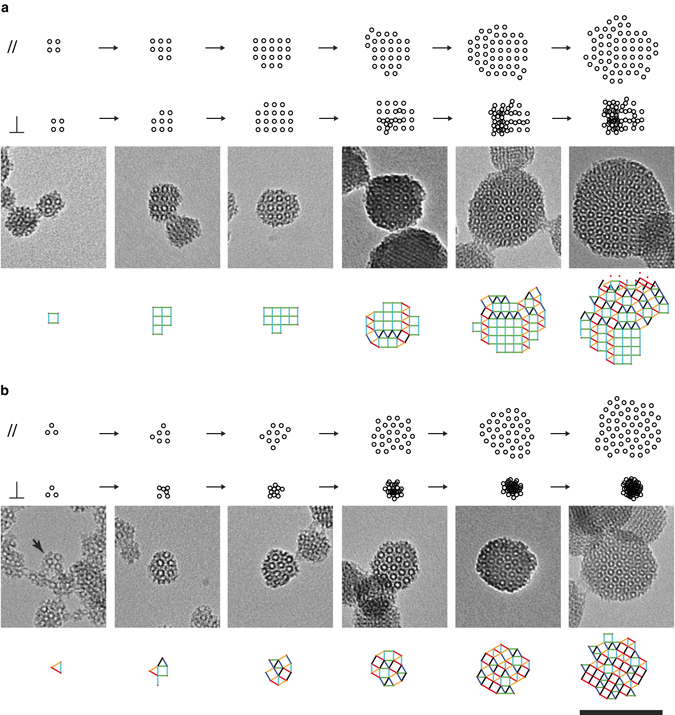
Comparison of simulated growth trajectories with TMOS/APTMS-derived MSNs. **a**, **b** Single-particle growth trajectories of two simulated particles with input triangle/square ratio equal to 0.1 with vertices plotted in both parallel and perpendicular space. Simulated growth stages are compared to experimental TEM images of TMOS/APTMS-derived MSNs and their tilings. Particles were synthesized under varying ammonium hydroxide and mesitylene concentrations as shown in Fig. 2e. The stirring rate was 600 rpm for all syntheses. All images have the same magnification; *scale bar* is 100 nm

Side-by-side comparison of Fig. 5b and c reveals that the first triangle addition seeds the growth of a quasicrystalline phase. While particle growth in real (parallel) space does not depend on the timing of the triangle addition, particle growth in perpendicular space is mostly terminated as soon as the first triangle is added. Single-particle growth trajectories (Fig. 6) of two simulated particles with the same triangle/square input ratio (0.1) illustrate this effect. Similarities between tilings from TEM images of particles synthesized at intermediate [TMB] and tilings of simulated particles at various stages of growth support the assumption that the formation of MSNs with dodecagonal tilings can be described using our model (Fig. 6).

## Discussion

We have discovered a series of four different structures in MSNs, including dodecagonal tiling, as a function of either pore expander concentration or stirring rate. Precise control of highly tunable silica sol–gel chemistry together with growth simulations has enabled identification of the early growth trajectories of quasicrystalline MSNs. Our results suggest that skewed micelle size distributions as mapped by cryo- TEM frustrate crystal formation and favor the growth of quasicrystalline structures. Our experiments further suggest that this skewness can be induced either by increasing amounts of micelle pore expander, or by simple increase of stirring rate. These results imply that size polydispersity of building blocks, rather than pH-dependent changes in interaction potentials between building blocks, as suggested earlier^6^, is the primary factor in triggering the growth of quasicrystalline mesoporous silica structures.

Our results suggest that due to fast silane condensation, quasicrystalline MSNs are likely formed via an irreversible micelle packing process. Although the micelles studied here are unlikely to undergo the local structural rearrangements that are observed in metal alloys during high temperature processing steps^14^, we expect that the simple synthetic control parameters we have identified can be translated to a wide variety of self-assembled systems. The accessibility of the experimental control parameters we introduce in this study may simplify the design of alternative self-assembly chemistries for studying quasicrystal growth, especially in systems of polymer micelles where addition of co-solvents and mechanical agitation have already been shown to affect building block polydispersity^34^. Finally, although we are limited by the silica chemistry to relatively small particle sizes, the experimental observation of quasicrystal formation via an irreversible growth mechanism may provide fundamental insights into the origin of quasicrystallinity in other materials systems.

## Methods

### Materials

CTAB (≥99%), ethyl acetate (EtOAc, ACS grade), ammonium hydroxide (NH_4_OH, 29%), 1,3,5-trimethylbenzene (TMB, 99%), AEAPTMS (95%), APTMS (95%), TEOS (≥99%), TMOS (≥99%), hydrochloric acid (HCl, 36.5–38%), acetic acid (glacial), and ethanol (absolute, anhydrous) were used as received without further purification. Deionized water (Milli-Q, 18.2 MΩ cm) was used throughout.

### Synthesis of TEOS/AEAPTMS-derived MSNs

TEOS/AEAPTMS-derived MSNs with different structures were synthesized in aqueous solution at room temperature through surfactant-directed silica condensation. Round bottom flasks and egg-shaped stir bars were used in all TEOS/AEAPTMS syntheses. MSNs were prepared with [TMB] ranging from 12 to 205 mM. EtOAc (0.44 mL), NH_4_OH (1.35 mL), and then TMB were added to an aqueous solution of CTAB (52.5 mL, 2.61 mM) stirred at 650 rpm. After 30 min, a mixture of TEOS (0.25 mL) and AEAPTMS (0.0375 mL) was added to the reaction under continued stirring and allowed to react for 5 min. Then, additional water (18.45 mL) was added and the reaction was left stirring for 24 h. On completion of the reaction, the particle suspension was neutralized with 2 M HCl, before cleaning by repeated centrifugation and redispersion in EtOH. CTAB was removed from the resulting particles by adding 5 vol% of acetic acid to the suspension and stirring for 30 min. Following CTAB removal, the particles were again cleaned by repeated centrifugation and redispersion in EtOH.

In order to study the effect of stirring rate on the structure of these particles, MSNs were synthesized using constant [TMB] (29 mM) and four different stirring rates: 500 rpm, 650 rpm, 800 rpm, and 1000 rpm. All other synthesis parameters were unchanged from the above description.

### Synthesis of TMOS/APTMS-derived MSNs

TMOS/APTMS-derived MSNs of different sizes and structures were synthesized by surfactant-directed silica condensation in room temperature, aqueous solution. Round bottom flasks and egg-shaped stir bars were used in all TMOS/APTMS syntheses. MSNs were prepared with [TMB] ranging from 4 to 116 mM and [NH_4_OH] ranging from 15 to 150 mM. High [TMB] resulted in more quasicrystalline particles and high [NH_4_OH] resulted in larger particle sizes. NH_4_OH and TMB were added to an aqueous solution of CTAB (10 mL, 22.78 mM) stirred at 600 rpm. After 2 h, a mixture of TMOS (34 µL) and APTMS (25 µL) was added to the reaction and allowed to react for 24 h under continued stirring. Applied synthesis conditions gave access to particle sizes between about 30 and 150 nm. On completion of the reaction, the particle suspension was cleaned by repeated centrifugation and redispersion in EtOH. CTAB was removed from the resulting particles by adding 5 vol% of acetic acid to the suspension and stirring for 30 min. Following CTAB removal, the particles were again cleaned by repeated centrifugation and redispersion in EtOH. For the APTMS study (Supplementary Fig. 4), the [NH_4_OH] was held constant at 120 mM and the APTMS concentration was varied from 0 to 14 mM. All other parameters were kept the same.

### Characterization

TEM images were taken using a FEI Tecnai T12 Spirit microscope operated at an acceleration voltage of 120 kV. FFT analysis was performed using ImageJ software. Each TEM sample was prepared after CTAB removal by evaporating 10 µL of suspension on TEM grid in dry air. Cryo-TEM images were collected under low-dose conditions using a customized FEI Titan Themis 300 operating at 300 kV equipped with a cryo-box and FEI Ceta 16 M camera. More details can be found in the Supplementary Methods.

SAXS patterns were obtained at the G1 station at the Cornell High Energy Synchrotron Source (CHESS) using a 10 keV beam and a sample-to-detector distance of 40 cm. All samples were powders prepared by vacuum drying MSN suspensions after CTAB removal and were imaged soon after drying.

Nitrogen sorption measurements were performed using a Micromeritics ASAP2020 instrument. For each measurement, approximately 10 mg of freshly vacuum-dried powder sample was degassed at room temperature under vacuum overnight prior to the analysis.

### Tiling analysis

Tilings were obtained from TEM images using a home-built MATLAB program, where a 10% tolerance of edge orientation and edge length were allowed and underdeveloped pores on MSNs were not counted. MSNs were excluded from analysis only if particle orientation prevented visualization of the pore structure from TEM images. No additional selection criteria were applied. These tilings were further analyzed using home-built programs to calculate radius of gyration in both parallel and perpendicular spaces. Detailed methods are provided in the Supplementary Methods.

### Simulations

An irreversible triangle-square tiling model was developed so that particle growth trajectories could be studied. Simulations were performed employing a self-written MATLAB program. See descriptions in the main text as well as more details in the Supplementary Methods.

### Data availability

The data that support the findings of this study are available from the corresponding authors on request.

## Electronic supplementary material

Supplementary Information
