# Supplementary material for: Formation pathways of mesoporous silica nanoparticles with dodecagonal tiling
Source: Nat Commun. 2017 Aug 15;8:252. doi: 10.1038/s41467-017-00351-8 (PMC5558005; doi:10.1038/s41467-017-00351-8)
Supplement: Supplementary file 1 — Supplementary Information [file 41467_2017_351_MOESM1_ESM.pdf]

Description of Supplementary Files

File Name: Supplementary Information
Description: Supplementary Figures, Supplementary Table, Supplementary Methods and Supplementary References

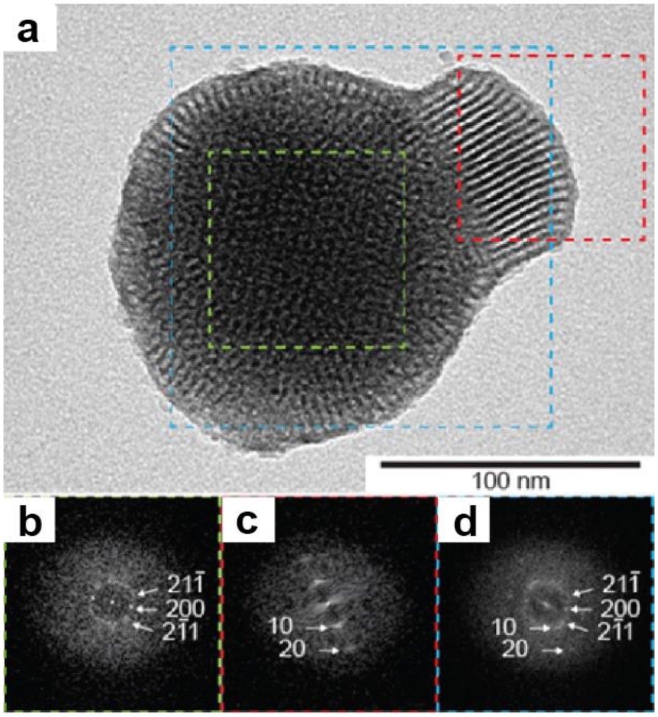

Supplementary Figure 1. Fast Fourier transform analysis of a tetraethyl orthosilicate/N-(2-aminoethyl)-3-aminopropyltrimethoxysilane derived multicompartment particle image

a, Transmission electron microscopy image of a multicompartment mesoporous silica nanoparticle with one branch, synthesized using 29 mM mesitylene and 13.8 mM ammonium hydroxide. The reaction stirring rate was 650 rpm. The scale bar is 100 nm. **b,** Fourier diffractogram (FD) of the cubic core region in (**a)** (green box), showing spots from the cubic $Pm\bar{3}n$ lattice along the [110] zone axis. **c**, FD of the hexagonal branch region in (**a**) (red box), showing spots from the hexagonal *p*6*mm* lattice. **d,** FD of the region containing both compartments in (**a**) (blue box), showing that the cubic 211 and hexagonal 10 spots reside closely with a lattice mismatch of 8.5% (larger for cubic lattice). This lattice mismatch is compensated by low-angle defects at the (111) plane.

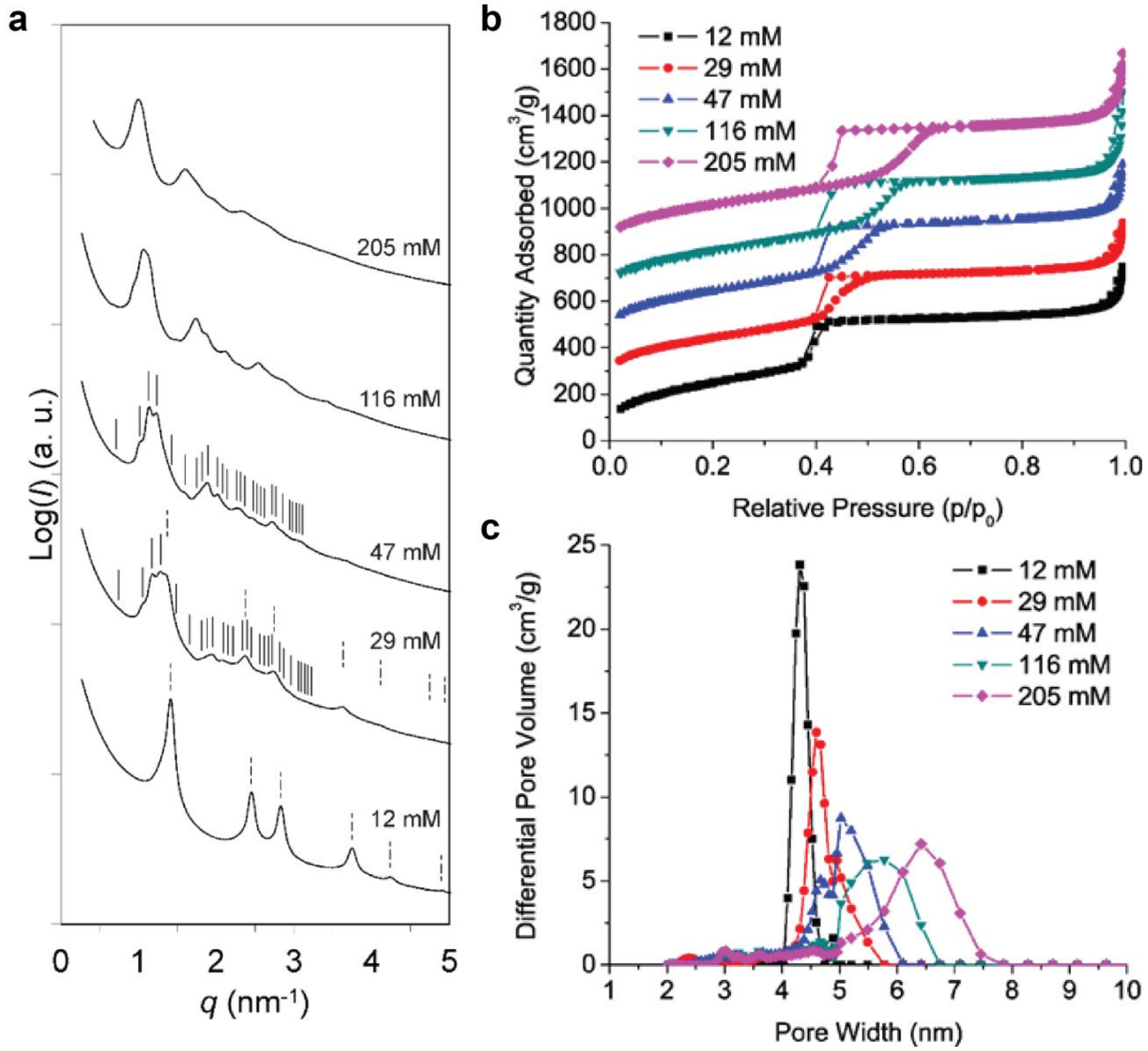

Supplementary Figure 2. Structure evolution of tetraethyl orthosilicate/N-(2-aminoethyl)-3-aminopropyltrimethoxysilane derived mesoporous silica nanoparticles as a function of mesitylene concentration as revealed by small angle x-ray scattering and nitrogen sorption

a, Small angle x-ray scattering patterns of mesoporous silica nanoparticles prepared at 12 mM, 29 mM, 47 mM, 116 mM, and 205 mM mesitylene (TMB). Particles were synthesized using constant ammonium hydroxide (13.8 mM) and stirring rate (650 rpm). Expected peak positions from cubic $Pm\bar{3}n$ and hexagonal $p6mm$ lattices are marked using solid and dashed lines, respectively. **b**, **c**, Nitrogen sorption isotherms (**b**) and corresponding density functional theory (DFT) derived pore size distributions (**c**) of mesoporous silica nanoparticles prepared at varying TMB concentrations. In (**b**), starting from the second curve, each isotherm is offset along the *y*-axis by 200 cm^3/g compared to the previous one. All samples exhibited type IV isotherms with increasing size of hysteresis loops, attributed to the expansion of mesopores, which was further elucidated by the pore size distributions calculated using a DFT model.

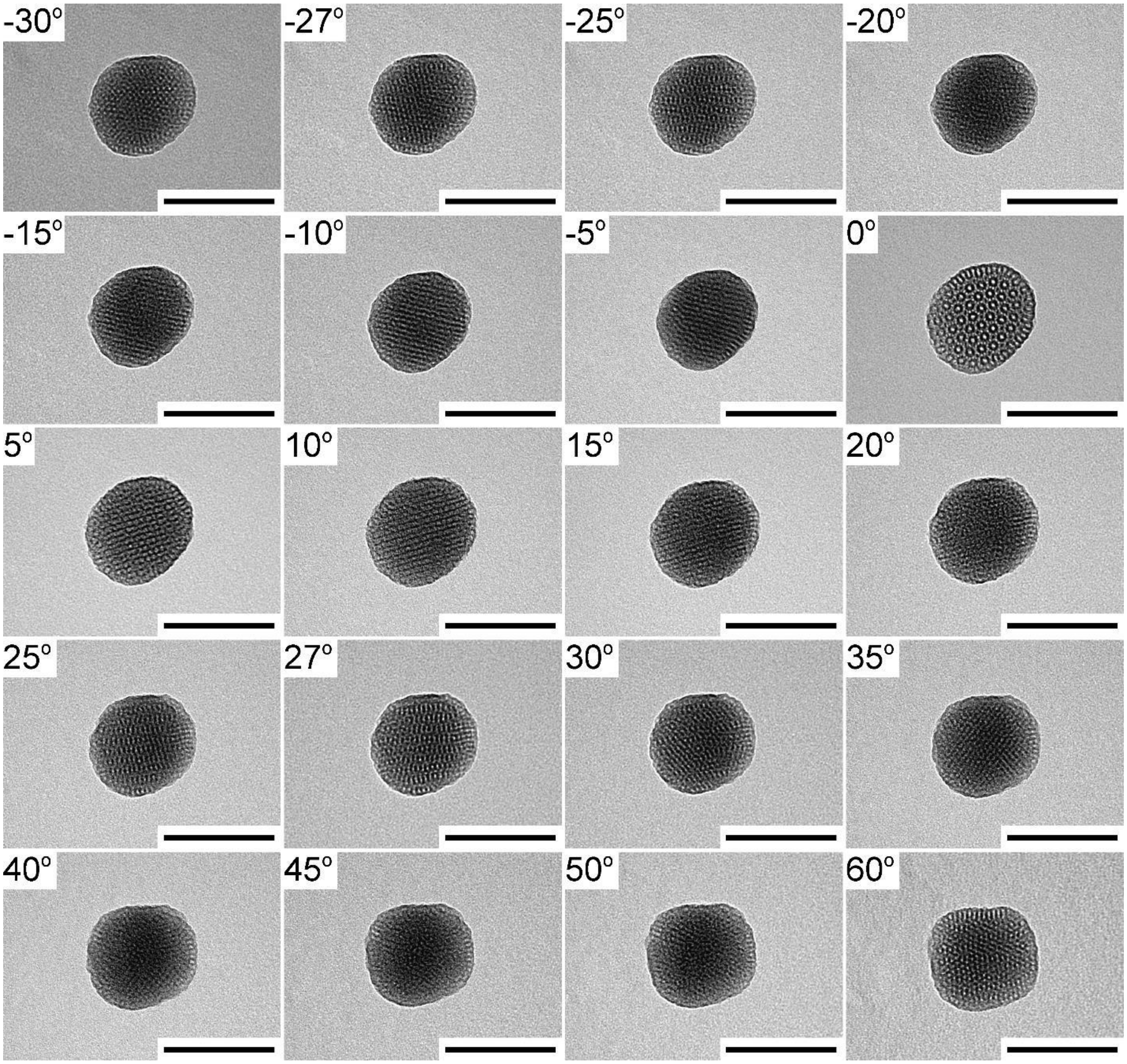

Supplementary Figure 3. **Single-tilt transmission electron microscopy study of a tetraethyl orthosilicate/N-(2-aminoethyl)-3-aminopropyltrimethoxysilane derived mesoporous silica nanoparticle with dodecagonal tiling**

Quasicrystalline tetraethyl orthosilicate/N-(2-aminoethyl)-3-aminopropyltrimethoxysilane particles were synthesized using 116 mM mesitylene and 13.8 mM ammonium hydroxide. The reaction stirring rate was 650 rpm. From these images it is difficult to extract the three-dimensional particle structure. All scale bars are 100 nm.

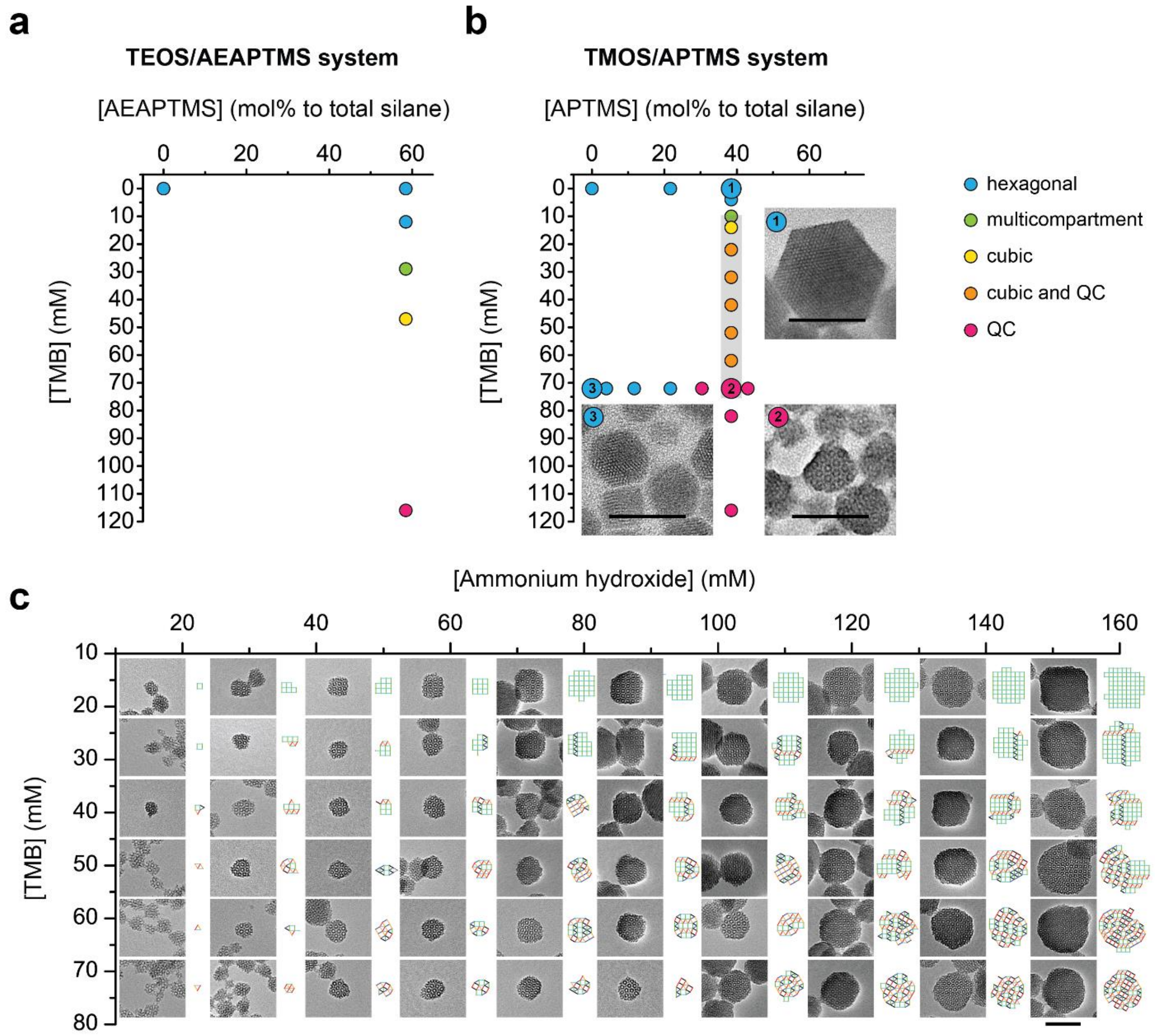

Supplementary Figure 4. Synthesis maps of mesoporous silica nanoparticles synthesized using two different silane systems

a, **b,** Structures of tetraethyl orthosilicate/N-(2-aminoethyl)-3-aminopropyltrimethoxysilane (TEOS/AEAPTMS) (**a**) and tetramethyl orthosilicate/(3-aminopropyl)trimethoxysilane **(**TMOS/APTMS) (**b**) derived mesoporous silica nanoparticles synthesized under varying mesitylene (TMB) and aminosilane concentrations. TEOS/AEAPTMS and TMOS/APTMS derived mesoporous silica nanoparticles were synthesized using 13.8 mM and 120 mM ammonium hydroxide, respectively. The insets in (**b**) are transmission electron microscopy (TEM) images of representative TMOS/APTMS derived particles. The numbered markers in the top left corner of each TEM image can be correlated to specific positions on the synthesis map. **c**, Extended data set relative to what is shown in Fig. 2e of the main text, including TEM images and square-triangle tilings of mesoporous silica nanoparticles synthesized using the TMOS/APTMS system. The concentration of APTMS used in synthesizing this series of particles was 14 mM. Scale bars in all images are 100 nm.

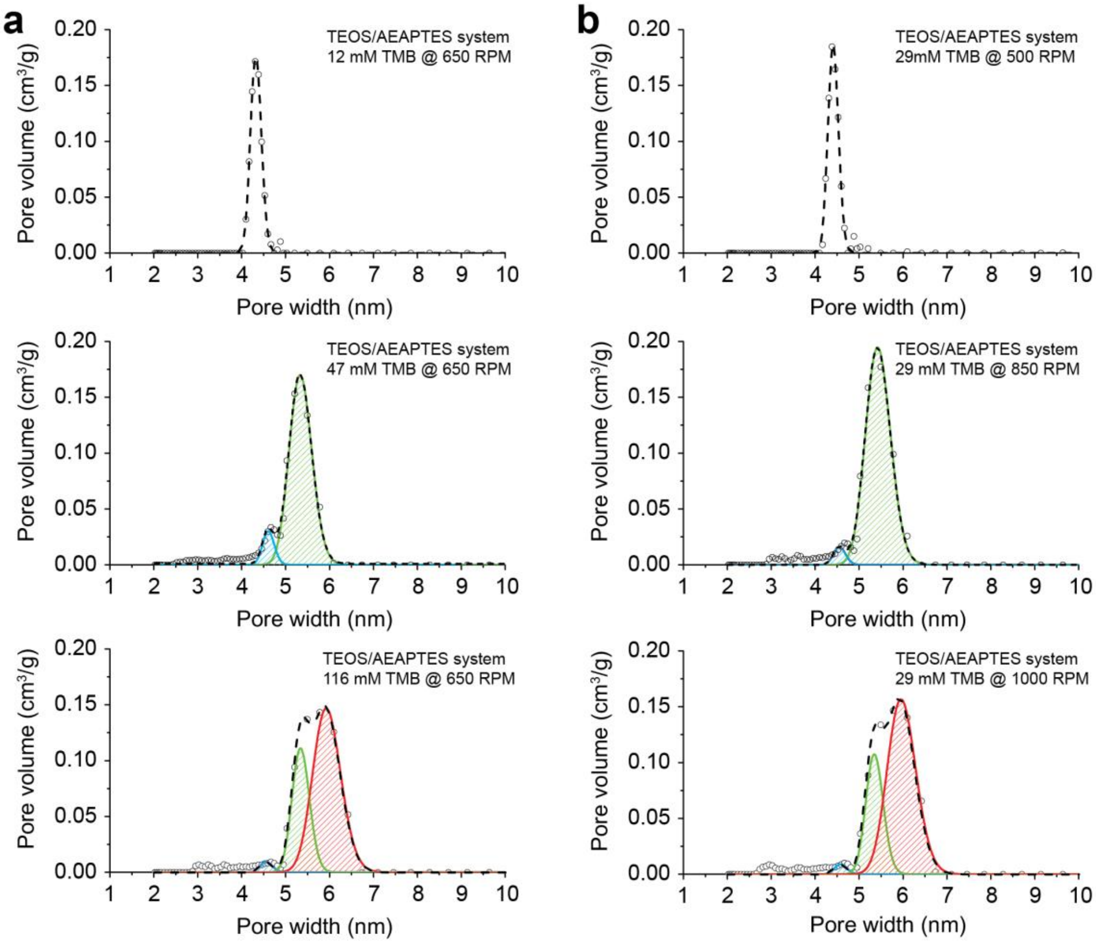

Supplementary Figure 5. Pore size analysis of tetraethyl orthosilicate/N-(2-aminoethyl)-3-aminopropyltrimethoxysilane derived mesoporous silica nanoparticles synthesized under varying conditions

a, Pore size distributions of mesoporous silica nanoparticles synthesized using the tetraethyl orthosilicate/N-(2-aminoethyl)-3-aminopropyltrimethoxysilane (TEOS/AEAPTES) system and varying mesitylene (TMB) concentrations. **b,** Pore size distributions of mesoporous silica nanoparticles synthesized using the TEOS/AEAPTES system and varying stirring rates. The ammonium hydroxide concentration in all syntheses was 13.8 mM. The pore size distributions obtained from fits of the corresponding nitrogen adsorption/desorption experiments shown in Supplementary Figures 2 and 6 were fit with linear combinations of one, two, or three lognormal functions. In bimodal and trimodal fits, small, medium, and large micelle distributions are colored blue, green, and red, respectively.

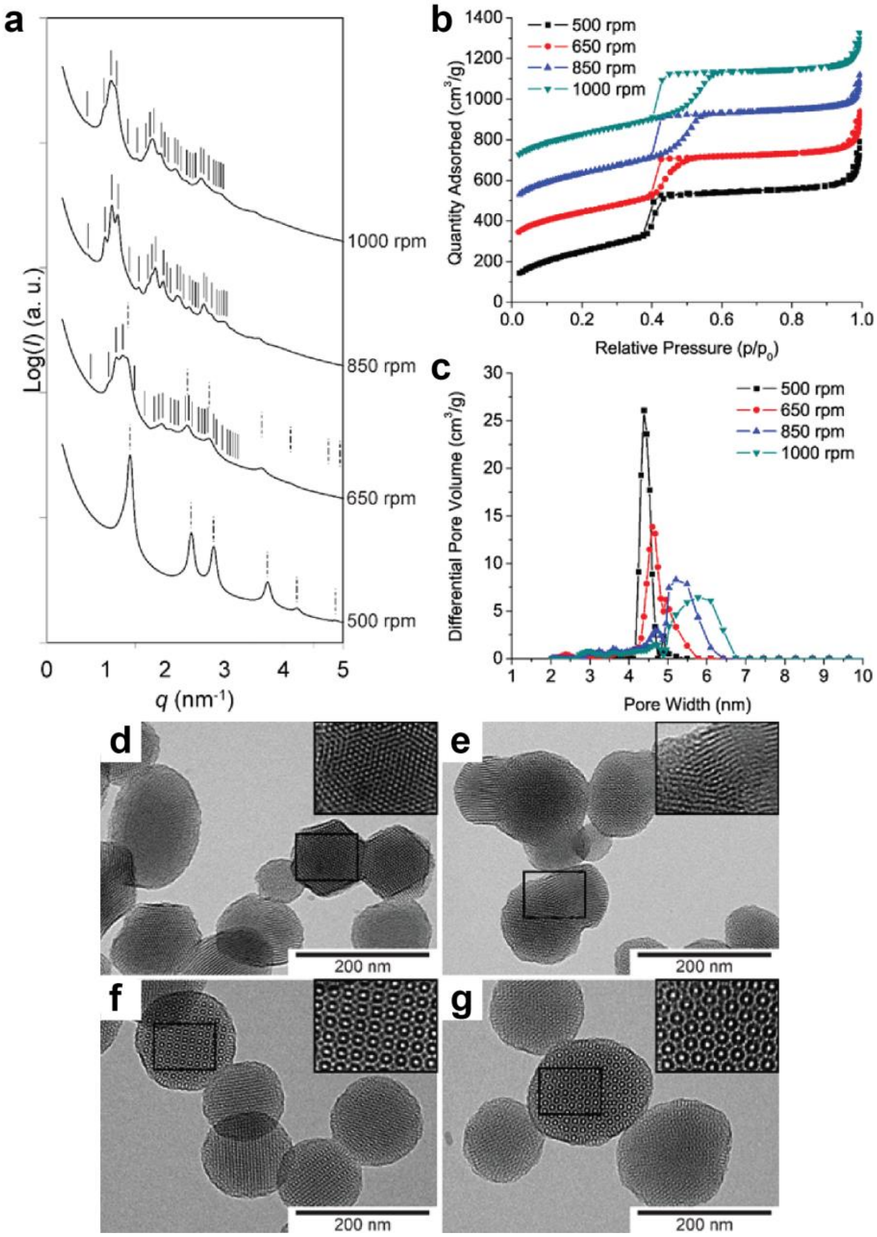

Supplementary Figure 6. Structure evolution of tetraethyl orthosilicate/N-(2-aminoethyl)-3-aminopropyltrimethoxysilane derived mesoporous silica nanoparticles as a function of stirring rate
(**a**) Small angle x-ray scattering (SAXS) patterns, (**b**) nitrogen sorption isotherms, and (**c**) corresponding density functional theory (DFT) derived pore size distributions of mesoporous silica nanoparticles prepared from tetraethyl orthosilicate (TEOS), N-(2-aminoethyl)-3-aminopropyltrimethoxysilane (AEAPTES), and 29 mM mesitylene (TMB) at varying stirring rates. The ammonium hydroxide concentration in all syntheses was 13.8 mM. In the SAXS patterns, expected peak positions for cubic $Pm\bar{3}n$ and hexagonal $p6mm$ lattices are indexed using solid and dashed lines, respectively. In (**b**), starting from the second curve, each isotherm is offset along the *y*-axis by 200 cm^3/g compared to the previous one. **d-g,** Transmission electron microscopy images of TEOS/AEAPTMS derived mesoporous silica nanoparticles prepared from 29 mM TMB at stirring rates of (**d**) 500 rpm, (**e**) 650 rpm, (**f**) 850 rpm, and (**g**) 1000 rpm. Scale bars are 200 nm. Insets are magnified images on selected areas (rectangles).

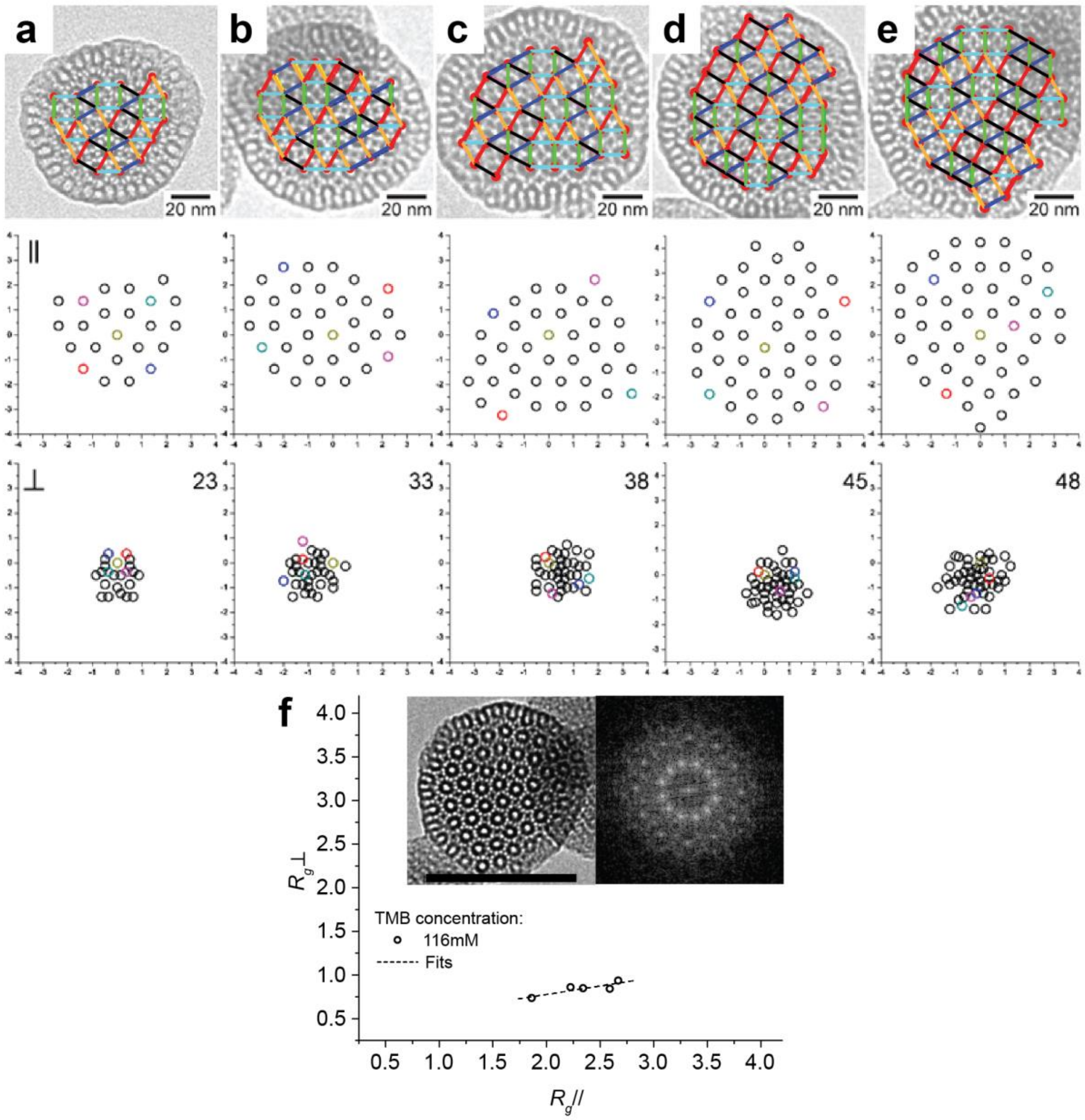

Supplementary Figure 7. Tiling analysis for mesoporous silica nanoparticles synthesized using the tetraethyl orthosilicate/N-(2-aminoethyl)-3-aminopropyltrimethoxysilane silane system at 116mM mesitylene

a-e, Square-triangle tilings superposed on high-magnification transmission electron microscopy (TEM) images of five dodecagonal mesoporous silica nanoparticles with (**a**) 23, (**b**) 33, (**c**) 38, (**d**) 45, and (**e**) 48 vertices. The randomly colored points in the associated parallel and perpendicular space plots show how individual points move during the transformation from parallel to perpendicular space. **f,** Corresponding phason strain analysis of mesoporous silica nanoparticles. Plot of R_g in perpendicular vs. parallel space, calculated for tetraethyl orthosilicate/N-(2-aminoethyl)-3-aminopropyltrimethoxysilane derived mesoporous silica nanoparticles synthesized at 116 mM mesitylene (TMB) and 13.8 mM ammonium hydroxide. The reaction stirring rate was 650 rpm. The phason strain determined from this analysis is 0.19. The insets show a TEM image of a quasicrystalline mesoporous silica nanoparticle (left) and the corresponding Fourier diffractogram (right), scale bar 100nm.

Supplementary Table 1. Pore size analysis derived from nitrogen sorption measurements on mesoporous silica nanoparticles synthesized using tetraethyl orthosilicate and N-(2-aminoethyl)-3-aminopropyltrimethoxysilane

Total pore volumes, average pore diameters, and full width at half maximum (FWHM) values are determined from log-normal fits of plots of incremental volume vs. pore diameter (fits shown in Supplementary Data Fig. 5).

**Varying [TMB] @ Constant Stirring Rate**					
**650 RPM**	**[TMB]**		**Small Pores**	**Medium Pores**	**Large Pores**
	**12 mM**	Total Vol. (cm^3/g)	0.055		
		Avg. Pore Width (nm)	4.3		
		FWHM (nm)	0.3		
	**47 mM**	Total Vol. (cm^3/g)	0.010	0.109	
		Avg. Pore Width (nm)	4.6	5.3	
		FWHM (nm)	0.3	0.6	
	**116 mM**	Total Vol. (cm^3/g)	0.003	0.055	0.117
		Avg. Pore Width (nm)	4.6	5.3	5.9
		FWHM (nm)	0.3	0.4	0.5
	**205 mM**	Total Vol. (cm^3/g)	0.002	0.028	0.186
		Avg. Pore Width (nm)	4.4	5.5	6.5
		FWHM (nm)	0.3	0.7	1.1
**Constant [TMB] @ Varying Stirring Rates**					
**29 mM TMB**	**Stirring**		**Small Pores**	**Medium Pores**	**Large Pores**
	**500 rpm**	Total Vol. (cm^3/g)	0.056		
		Avg. Pore Width (nm)	4.4		
		FWHM (nm)	0.3		
	**850 rpm**	Total Vol. (cm^3/g)	0.005	0.139	
		Avg. Pore Width (nm)	4.6	5.4	
		FWHM (nm)	0.3	0.7	
	**1000 rpm**	Total Vol. (cm^3/g)	0.003	0.053	0.127
		Avg. Pore Width (nm)	4.6	5.4	6.0
		FWHM (nm)	0.3	0.5	0.8

Supplementary Methods

Cryo-TEM and micelle size measurements

For direct observation of micelle size and size distribution, five samples of native micelle solution (before silane addition) with varying [TMB] and stirring conditions were subjected to cryo-TEM characterization. The first four samples, containing 0mM, 4mM, 72mM and 116mM TMB, were prepared by adding TMB into CTAB/NH_4OH (22.78mM/150mM) aqueous solution under stirring at 600 rpm. The stirring was maintained at 600 rpm for two hours before an aliquot of the micelle solution was removed for cryo-TEM sample preparation. Immediately afterward, TMOS and APTMS were added to the native micelle solutions to make the corresponding MSNs shown in the bottom row of Fig.3a through d. The fifth sample, containing 72mM TMB, was prepared by adding TMB into CTAB/NH_4OH (22.78mM/150mM) aqueous solution under stirring at 600 rpm. The stirring was maintained at 600 rpm for about 30 seconds to ensure homogeneous dispersion of TMB into the solution. The stirring was then stopped and the solution was left static for two hours before cryo-TEM sample preparation. Then, the stirring was started again and maintained at 100 rpm during TMOS and APTMS addition. One minute after silane addition, the stirring was stopped and the reaction solution was kept static through the remainder of the synthesis. TEM results of this synthesis are shown in Figs. 3e, j, and o.

Samples for cryo-TEM analysis were prepared by plunge freezing. 4 μL of solution were pipetted onto a Quantifoil holey carbon TEM grid that was plasma cleaned for 10s with a mixture of argon and oxygen gases. The specimen was manually blotted for 6-8 seconds and plunged into a liquefied mixture of ethane and propane[1]. Grids were transferred to the TEM and imaged below the recrystallization temperature of the specimen using a Gatan cryo-transfer holder. Cryo-TEM images were collected under low-dose conditions using a customized FEI Titan Themis 300 operating at 300 kV equipped with a cryo-box and FEI Ceta 16M camera. All images were collected at -2 μm defocus at a dose between 80-120 electrons/$Å^2$.

Micelle size distributions were determined from cryo-TEM images. Images were cropped to regions of micelles suspended in vitreous ice in a TEM grid hole. Using in-house python code, micelles were located via a Laplacian of Gaussian blob-detecting algorithm and their sizes measured by fitting the local image intensity at the location determined by the blob-finder to a two-dimensional Gaussian function. Reported diameters are $2\sqrt{2}\sqrt{\sigma_x^2 + \sigma_y^2}$, where σ_x and σ_y are the standard deviation of the 2D Gaussian in x and y directions determined by the longest and shortest axes of the micelle. For each image, the intensity threshold of the blob-detecting algorithm was set by hand to ensure correct identification of micelles without inclusion of noise. Size distributions for each condition were calculated from 5-7 images collected at different locations across the TEM grid.

To describe the shape of the micelle size distributions, the histograms were fit to a skew-normal distribution using nonlinear least squares fitting in python:

$$A\left(1 + \mathrm{erf}\left(\frac{\alpha(x-\mu)}{\sigma\sqrt{2}}\right)\exp\left(-\frac{(x-\mu)^2}{2\sigma^2}\right)\right), (1)$$

where erf is the error function, A, μ and σ are amplitude, average size and scale parameter, respectively. The parameter α describes the departure from the normal distribution and improves

fitting for distributions with a longer tail. The skew-normal distribution was chosen because it describes well the tail of the size distribution contributed by larger micelles[2].

Tiling Analysis

The edges of the square-triangle tiling correspond to the six dodecagonal directions in physical (or parallel) space. Each vertex in parallel space can be expressed as

$$\mathbf{r}^{\|} = n_1\mathbf{e}_1^{\|} + n_2\mathbf{e}_2^{\|} + n_3\mathbf{e}_3^{\|} + n_4\mathbf{e}_4^{\|}, \quad (2)$$

where n_i is a unique set of integer coordinates and $\mathbf{e}_i^{\|} = \{\cos(\alpha(i - 1)), \sin(\alpha(i - 1))\}$ with $\alpha = \pi/6$ and $i = 1\text{-}4$. The corresponding vector $\mathbf{r}^{\perp}$in perpendicular space is

$$\mathbf{r}^{\perp} = n_1\mathbf{e}_1^{\perp} + n_2\mathbf{e}_2^{\perp} + n_3\mathbf{e}_3^{\perp} + n_4\mathbf{e}_4^{\perp}, \quad (3)$$

where n_i is again a unique set of integer coordinates and the dodecagonal directions in perpendicular space are $\mathbf{e}_i^{\perp} = \{\cos(7\alpha(i - 1)), \sin(7\alpha(i - 1))\}$ with $\alpha = \pi/6$ and $i = 1\text{-}4$. As shown in Figure 2d, the 2D parallel and perpendicular spaces are identified by means of consistency of $\mathbf{e}_1^{\|,\perp}$ and $\mathbf{e}_3^{\|,\perp}$.

Following transformation from parallel to perpendicular space, a crystalline pattern, e.g. with pure square or triangle packing, remains unchanged. In contrast, a quasicrystalline pattern shrinks upon translation to perpendicular space. The degree of shrinkage reflects the disorder in the triangle-square packing and can be used as a measure of quasicrystallinity. For this reason, the quasicrystallinity of a pattern can be obtained by comparing the radii of gyration of the patterns in parallel and perpendicular spaces. The relationship between the radii of gyration in the two spaces can be described by the linear equation $R_g\perp = AR_g// + B$, where the slope provides the magnitude of the phason strain, a quantitative measure of quasicrystallinity. The phason strains of an ideal quasicrystal and crystal are 0 and 1, respectively.

Tilings of the synthesized MSNs were obtained from TEM images using a self-written MATLAB program, where 10% tolerance of edge orientation and edge length are allowed and undeveloped pores were not counted. These tilings were further analyzed by using a second self-written program to calculate $R_g//$ and $R_g\perp$ and to quantify the presence of the four fundamental geometrical conformers present in the structure, i.e. 4^4, 3^34^2, 3^2434 and 3^6 (see details in main text). Considering that differently sized MSNs synthesized at the same [TMB] but at varying pH are expected to have similar micelle size and size distribution, a linear fit ($R_g\perp = AR_g// + B$) was applied to MSNs synthesized at the same [TMB]. For MSNs synthesized at the highest [TMB] with the highest quasicrystallinity, the plot of $R_g\perp$ vs. $R_g//$ was described well by this linear relationship. This suggests that individual MSNs have the same type of pattern, and thus the fitted slope represents the phason strain of the structure obtained from this [TMB]. In contrast, as [TMB] decreased, large particles synthesized at intermediate [TMB] exhibited substantial fluctuations in the plot of $R_g\perp$ vs. $R_g//$, suggesting that the MSN structure obtained from those synthesis conditions was heterogeneous. Therefore, in this case the slope of the linear fit does not reflect the phason strain of these individual particles. However, the fact that the slope of these

fits decreased with increasing [TMB], suggests that the average quasicrystallinity of all the MSNs from a single synthesis is highly dependent on [TMB].

Growth Simulations

Growth of quasicrystalline mesoporous silica materials has previously been modeled as a random aggregation of triangle and square tiles[3]. The relationship between these fundamental triangle/square units and CTAB micelle packings is illustrated schematically in Figure 1m. While these simulations do not directly address the details of the micelle packing process, they can play an important role in developing an understanding of the structural arrangement of triangle/square tiles observed experimentally in quasicrystalline mesoporous silica. We have therefore modeled our experimental system using a similar tiling process[3].

We employed a random square-triangle tiling process wherein cluster growth, seeded by an individual equilateral triangle or square tile, proceeds by sequential and irreversible edge-to-edge addition of triangle and square tiles with edge length *a*. The seed tile and all subsequent tiles were randomly chosen from a weighted distribution defined by an input triangle/square ratio that reflects the micelle size distribution at varying [TMB]. New tiles were placed at random, according to a probability-weighted list of candidate growth sites defined as edges that lie on the boundary of the growing cluster. Candidate sites were assigned probabilities according to physically inspired interaction potential energies. The tiling process proceeded until the final particle size, determined at the start of each simulation from a weighted normal distribution about an average particle size, was reached. Importantly, we did not make any effort to suppress the stochastic noise that accompanies this process. Comparison with experimental data showed that these results may reflect local fluctuations of the micelle sizes in solution.

In defining interaction potentials for candidate growth sites, we included two separate contributions, one that reflects the incoming shape and the geometry of the site and one that is directly related to the distance, *d*, of the site from the center of mass of the growing cluster. Interaction potentials were optimized so that an input triangle/square ratio of 2.3 (chosen to reflect the triangle/square ratio in an ideal dodecagonal quasicrystal) produced clusters with quasicrystalline features and were then held constant for the remainder of these simulations. The form of the attachment probability, *P*, associated with each candidate site was also considered a fitting parameter and was ultimately determined to be:

$$P = P_s \times (d/d_0)^{-10}, \quad (4)$$

where P_S is the attachment probability associated with the surface site geometry, and *d*, the distance from center parameter is normalized by d_0, the shortest radial distance from the particle edge to its center of mass. This surface minimizing condition produced tilings that mirror the spherical morphology of particles observed in experiments. Contributions to P_S are specified as follows:

(1) The edge-edge contact between a square and a triangle P_{ST}, two triangles P_{TT}, two squares P_{SS}.
(2) The interaction between an incoming square tile and an incomplete 90º corner, P_R.

(3) The interaction between an incoming triangle tile and incomplete 60º or 120º corners, P_T .

Using a triangle/square ratio of 2.3, parameter values were $P_{ST} = 0.75$, $P_{TT} = 0.001$, $P_{SS} = 0.001$, $P_R = 0.75$, and $P_T = 0.75$. We found that it was necessary to include P_{TT} and P_{SS} as constant repulsive parameters to prevent the formation of large hexagonal or cubic domains, which were not observed in our experimental particles. These repulsive parameters may reflect interfacial interactions between micelles[3]. Additionally, P_R and P_T were included as attractive parameters to discourage defect formation. For sites that could be assigned to multiple categories, the highest P_S was used. However, if tile attachment generates a defect that cannot accommodate either shape, $P_S = 0.0000075$. Thus each growth site was given a single interaction potential, specifically defined as the probability that particular site will be filled. For each tile addition, a revised list of interaction potentials was generated to reflect changes to the list of candidate growth sites and a specific growth site was randomly chosen from this probability-weighted list. Optimized values of P_S were kept constant for the remainder of the simulations where the triangle/square number ratio was varied to reflect the range of [TMB] used in experiments.

In order to address the possibility that only growth sites with very low attachment probabilities exist, an additional threshold parameter was included. This attachment threshold, T, is given by

$$T = 0.000075 \times r, \quad (5)$$

where r is the maximum radial distance from the particle edge to its center of mass. If the growth site chosen from the distribution of attachment probabilities is below T, then the tile is not placed. Instead, a new tile is chosen from the shape distribution and the choice is repeated. If after 100 attempts, no site with $P > T$ is chosen, then the growth site is temporarily removed from the list of candidate sites and a new tile is again chosen. This process is repeated until a tile can be placed and growth can proceed. Since the attachment probability is shape specific, the threshold mechanism may act as a barrier to attachment of either type of tile, allowing mismatches between the input triangle to square ratio and resulting particle composition.

For each triangle/square ratio, four batches of particles with varying average diameter were generated and combined for analysis. Final particle size was determined at the start of each simulation by random choice from a probability weighted distribution about an average size. The simulation was terminated when the particle reached the predetermined size. In defining the distribution of particle sizes, we utilized a set of four normal distributions with mean (μ) and variance (σ) parameters as follows.

Set A: $(\mu, \sigma) = (1.5, 0.5)$

Set B: $(\mu, \sigma) = (3.5, 1.5)$

Set C: $(\mu, \sigma) = (6.0, 2.0)$

Set D: $(\mu, \sigma) = (7.5, 2.5)$

These distributions were chosen to reflect the sizes of the particles observed in experiments.

Fitting of the relationship between $R_g\perp$ and the total number of squares before the first triangle is attached

The black dots in Figure 5b and c, for which no triangle ever entered the simulations, represent the relationship between the total area and the radius of gyration of a 2D square packing. However, it is interesting to note that all the other data points in Figure 5c, which came from the simulations whose final structure does contain triangles, lie on the same curve. This indicates that the growth of particles in perpendicular space follows the same trend whether or not a triangle is involved in the particle growth; and this is independent of the particle's final quasicrystallinity. In order to quantitatively resolve this general relationship, all the data in Figure 5c were fitted using the equation $N = N\,(R_g\perp)$ of 2D tiling of squares. To derive the fitting equation, an approximation was first made that the shape of the overall tiling is a perfect square. This is consistent with experiments in which cubic MSNs always have square facets (Fig. 1d).

According to the definition of the radius of gyration, R_g, the radius of gyration of a 2D square packing follows the equation:

$$R_g{}^2 = \frac{1}{\left(\sqrt{N}+1\right)^2}\sum_{n=0}^{\left(\sqrt{N}+1\right)^2} r_n{}^2, \quad (6)$$

where N is the total number of squares and r_n is the distance of point n to the center of mass of the packing:

$$r_n = a \cdot \sqrt{\left(x_n{}^2 + y_n{}^2\right)}, \quad (7)$$

where x_n and y_n are the normalized coordinates of point n in the coordinate system whose origin is the center of mass of the overall square tiling. $a = 1$ is the edge length of a single square tile.

R_g can then be expressed as:

$$\left(\frac{R_g}{a}\right)^2 = \frac{1}{6}\left(\left(\sqrt{N}+1\right)^2 - 1\right), \quad (8)$$

Solving for N yields the relationship between R_g and N:

$$N = 6\left(\frac{R_g}{a}\right)^2 - 2\sqrt{6\left(\frac{R_g}{a}\right)^2 + 1} + 2, \quad (9)$$

N for large packings, where $6\left(\frac{R_g}{a}\right)^2 \gg 1$, can be approximated by:

$$N \approx 6\left(\frac{R_g}{a}\right)^2 - 2\sqrt{6}\left(\frac{R_g}{a}\right) + 2 \approx 6\left(\frac{R_g}{a}\right)^2 - 4.9\left(\frac{R_g}{a}\right) + 2, \quad (10)$$

Since $a = 1$,

$$N \approx 6R_g{}^2 - 4.9R_g + 2, \quad (11)$$

We therefore fitted the data in Figure 5c using the equation:

$$N = AR_g\perp^2 + BR_g\perp + C, \quad (12)$$

where the fitted values A, B and C were equal to 6.3, -6.3 and 2.6, respectively. These values agree well with the theoretical expectations as described above.

Supplementary References

1. Tivol, W. F., Briegel, A. & Jensen, G.J. An Improved Cryogen for Plunge Freezing. *Microsc. Microanal.* **14,** 375-379 (2008).
2. Azzalini, A. A class of distributions which includes the normal ones. *Scand. J. Stat.* **12**, 171-178 (1985).
3. Xiao, C., Fujita, N., Miyasaki, K., Sakamoto, Y. & Terasaki, O. Dodecagonal tiling in mesoporous silica. *Nature* **487**, 349-353 (2012).
